# Self-Testing for Dementia: A Phenomenological Analysis of Fear

**DOI:** 10.1007/s10912-024-09849-x

**Published:** 2024-06-12

**Authors:** Alexandra Kapeller, Marjolein de Boer

**Affiliations:** 1 Department for Thematic Studies: Technology and Social Change, Linköping University, Temahuset, 58330 Linköping, Sweden; 2 Department of Culture Studies, Tilburg University, PO Box 90153, 5000 LE Tilburg, The Netherlands

**Keywords:** Phenomenology, Dementia, Mild neurocognitive disorder, Fear, Self-testing apps, mHealth

## Abstract

Following the growing economic relevance of mobile health (mHealth) and the increasing global prevalence of dementia, self-testing apps for dementia and mild neurocognitive disorder (MCD) have been developed and advertised. The apps’ promise of a quick and easy tool has been criticized in the literature from a variety of angles, but as we argue in this article, the celebratory characterization of self-testing also stands in disbalance to the various kinds of fears that may be connected to taking the test. By drawing on Sara Ahmed’s phenomenological theory on emotions and by referring to illustrative experiences from two users with a particular dementia self-testing app, we explore four dimensions of fear derived from phenomenology: performative, ontological, embodied, and temporal dimensions. We argue that fear (1) motivates one to take the self-test and to try to take control over one’s health; (2) is shaped by and shapes the ways in which we make sense of ourselves and others as cognitively deficient; (3) constructs and is constructed by our differently embodied presence in the world; and that (4) testing makes a fearful future self as cognitively deficient more tangible. In outlining these different dimensions of fear, this article expands the understanding of the meaning of experiencing self-testing in comparison to the mostly quantitative literature on this topic.

## Introduction

In the context of dementia, self-testing has been presented as a potential tool to increase low diagnostic rates (Charalambous et al. 2020; Visser et al. 2021). Globally, the number of people at risk of getting dementia or mild neurocognitive disorder (MCD) is estimated to triple from 2015 to 2050 (Prince et al. 2016). In light of this public health challenge, it has been argued that early detection is paramount for timely, efficient, and cost-effective care (Ashford et al. 2007; Swan 2012). Digital (self-)testing for dementia—for example, in the form of apps that provide “quick and easy” testing in a few minutes at home—could lower the threshold of getting tested and thereby contribute to such early detection (Ruggeri et al. 2016). Moreover, self-testing apps, like other mobile health (mHealth) apps, have been advertised with the term *empowerment* (Kapeller and Loosman 2023), which means providing people with the skills and knowledge necessary to take (more) responsibility for setting and reaching their health goals (Funnell et al. 1991; Morley and Floridi 2020, 1166; Roberts 1999). mHealth apps could enable users to get tailored support and counseling in the doctor’s office (Janssen et al. 2020) and overall better care (Alexander and Joshi 2016; Pai et al. 2018; Swan 2012, 98).

Besides these celebratory voices about such mHealth solutions, there are many critiques of using more self-testing apps. This article contributes to them by engaging with self-testing apps in relation to the topic of fear. Some previous critiques center on the supposed increased worry, insecurity, and particularly fears of users when they interpret the test results (den Oudendammer and Broerse 2019). It has even been argued that screening practices, to which self-testing could be added, would perpetuate a *culture* of fear about health (Rosenberg 2009). Several studies in medicine, psychology, and cultural studies have connected fear to either self-testing (Kumwenda et al. 2018; Mollema et al. 2001) *or* dementia (Cipriani and Borin 2015; Kessler et al. 2012; Page et al. 2019). However, fear in specifically *self-testing for dementia* has not hitherto been addressed. While the predominantly quantitative (psychological and medical) studies about fear in (the context of) either dementia or self-testing are valuable in discerning who is fearful and in categorizing various aspects of fear, they do not provide in-depth analyses and understanding of what this fear may entail and how it may shape users’ experiences of the test, themselves, and their lives. This research lacuna is unfortunate because a richer conceptualization of fear in this context can further rebut the idea of a “quick and easy” test and may contribute to improving apps and healthcare practices so that they induce less fear. It would also contribute to understanding the underexplored “complexities and ambivalences that are part of using self-monitoring and self-care technologies for monitoring health and illness states” (Lupton 2013).

In this article, we explore what fear in relation to self-testing for dementia may mean for users’ experiences by building on Sara Ahmed’s phenomenology of fear (Ahmed 2003; 2015) and on the experiences of two people using a dementia self-testing app. In doing so, we do not provide a systematic qualitative study of lived experiences of self-testing but rather show how a phenomenological, theoretical analysis illustrated by two user interviews offers a more in-depth understanding of fear in self-testing in comparison to existing studies on these kinds of topics. Moreover, by focusing on the *experience* of using the self-testing technology, this article is not so much about the materiality of the technology or what the technology affords in its design while using it (Lupton 2019). By focusing on lived user experiences, our phenomenological article may help to evaluate the meaning of everyday dealings with self-testing apps in dementia diagnosis.

## Introducing the Case of a Dementia Self-Testing App

### The App

Our case is a North American dementia self-testing app that has been downloaded over 100,000 times.^1^ Like many other testing apps on the market, it is a digital version of an established pen-and-paper test that detects early signs of cognitive, memory, or thinking impairments. We decided on this app because it has been scientifically validated^2^ and because it is, according to our research, the only widely used dementia self-testing app on the market.

When starting the app, users are first presented with an introduction video that explains what mild cognitive impairment is, why early detection is important, and how the test works. Users are then asked to provide credit card details (the app charges after a free trial) and to find a “comfortable and quiet environment” for taking the test. The test itself includes tasks that test memory, problem-solving skills, and language abilities. For example, users should fill out the date and day of the week, name two pictures, complete a trail-making test, and draw a clock. These tasks can be done in 15 minutes. After a few days, users receive a file with their result, a numerical score, which is also explained in a short, personalized video. Importantly, the result does not constitute a diagnosis but is presented as an indication of whether seeking professional help and further testing at a doctor’s office may be necessary.

### The Interviewees

Apart from the direct-to-consumer (DTC) model, the app can also be used in health practices. To illustrate our theoretical findings, we used interviews with two older adults who had taken the test in such circumstances: in a doctor’s office, unsupervised. Both interviewed persons were white, male, well-educated, and 80 years old. Participants were recruited via their doctor, who was involved in the app’s development and who informed them about our research after they had received their test results. One participant took the test as a routine check-up, while the other took it because others had voiced concerns about his cognitive health. The doctor informed them that the test would constitute an indication of whether further testing was necessary and suggested that instead of traditional pen-and-paper, they could take the test on the app. The interviews with these two users were conducted online, over Zoom, and took 30 and 80 minutes. They were audio-recorded and transcribed ad-verbatim. Ethics approval for these semi-structured, qualitative interviews was granted by the relevant ethics boards,^3^ and participants gave their written consent^4^ before the interviews took place. The data were then analyzed through an interpretative phenomenological research method (Smith, Flowers, and Larkin 2009). Additionally, as we will elaborate later, phenomenological theories on fear were used to interpret the empirical data (see Ahmed 2003, 2004, 2015).

## Previous Research on mHealth and Fear

Self-testing apps, like our case, fall into the category of mHealth devices (Jutel and Lupton 2015; Lupton and Jutel 2015). But unlike mHealth applications such as fitness trackers or disease monitoring apps, self-testing apps provide information neither to people who have not been diagnosed with the respective disease and want to improve their lifestyle nor to people who have been diagnosed already and use apps alongside in-person healthcare. Instead, they provide potentially diagnostically relevant information to users who suspect they have a certain disease or want confirmation that they do not. Importantly, self-testing apps offer this information outside of a doctor-patient relationship, which might be necessary for contextualization and support (Kapeller and Loosman 2023). They can be seen as paradigmatic for positioning individuals as “digitally engaged patients” who can control their bodies and take responsibility for their health (Lupton 2013).

Positioning the user as responsible, self-sufficient, and in control is questionable when examining the various fears that a user may experience in relation to self-testing for dementia, a stigmatized and mostly incurable disease. To our knowledge, no studies on the experience of dementia self-testing have been conducted so far. Instead, we can draw on existing literature on experiences with other home-based, mostly non-digital, self-tests such as for COVID-19, HIV, or HPV. In categorizing different kinds of fear and outlining when people experience fear in the testing process, these studies are valuable in sociologically mapping fear in self-testing (i.e., in broadly defining fear and describing how it may function). For example, users are afraid of making a mistake during the test and getting the wrong result (Sarkar et al. 2016), and fear may constitute a consequence of receiving an unwanted result or as a motivator to take the test (McFarlane, Morgan, and Schlumbrecht 2021). These studies, however, do not focus on conceptualizing and examining this experience in-depth. More starting points can be identified from the literature on fear and dementia in general. Fear of cognitive disorders is widespread (Kessler et al. 2012, 276). It takes shape as a fear of discrimination by employers or healthcare problems (Stites, Rubright, and Karlawish 2018, 6), a fear of social embarrassment and long-term dependency (Husband 2000, 544), a fear of losing independence, control, identity, and dignity (Corner and Bond 2004, 150), and a fear of getting upset or depressed, or even a fear of dying by suicide once one receives a diagnosis (Van Den Dungen et al. 2014, 1614).

All these fears may come together in the digital practice of dementia self-testing. As technologies, self-testing apps mediate their user’s relation with their environment (Verbeek 2005), including cultural images of dementia, stigma, and the responsibility to test. In doing so, they may bundle the fears of using the test incorrectly, having a stigmatized, incurable disease, and being dependent and poor. Moreover, self-testing apps may also increase such a culture of fear as they are more accessible than traditional, paper-based self-tests and may be taken in the privacy of one’s personal life without proper guidance to elevate possible fears. Self-testing apps, furthermore, may also cause specific kinds of fears related to insecurity about using digital devices or even technophobia.

In order to explore the possible meaning(s) of fear in dementia digital self-testing for users’ experiences, we build on a philosophical phenomenological perspective to analyze and interpret the interviewees’ narrations about using a self-test app for dementia. Phenomenology allows for an in-depth, complex analysis of fear in dementia self-testing by emphasizing the centrality of situatedness in understanding people’s experiences and emotions and showing how such contextualized experiences may have repercussions for people’s self-understandings and identity. That is, people’s ways of experiencing—in this case—fear, dementia, and self-testing for dementia would be shaped within and through the mode and limits of the lived contexts in which they find themselves: the relationships they have; the (normative) culture(s) that they live in; their in/capable bodies; their memories, wishes, and hopes; and, of course, the things or technologies that they interact with (de Boer 2016; Merleau-Ponty [1945] 1962). People’s socio-cultural, embodied, temporal, and material context, in this sense, is the *sine qua non* for the ways in which we experience things, others, and of course, ourselves. Phenomenology, in other words, teaches us that contextualized experiences condition and shape the ways in which we (are able to) understand ourselves as selves; they co-construct one’s identity work and identity.

## Interpretative Framework: Phenomenology of Fear

Sara Ahmed’s phenomenology of emotion (Ahmed 2003; 2004; 2015) has been used to analyze fear in medical contexts before (Guntram and Zeiler 2016, 66) and can be instrumental for analyzing the experience of self-testing for dementia. In her book *The Emotional Politics of Emotion* (2015), Ahmed is concerned with the ontological *performativity* of emotions. Rather than focusing on the ontology of emotions—asking, “What are emotions?”—she asks, “What do emotions do?” (Ahmed 2015, 4). In answering this question, she critiques what she calls the psychological model, which conceptualizes emotions as internal individual dispositions, and the social science model, which conceptualizes emotions as the cohesion of social bodies or collectives (Morrison 2020, 151).

Ahmed argues that both models “assume the objectivity of the very distinction between inside and outside, the individual and the social, the ‘me’ and the ‘we’ ” (Ahmed 2015, 9). She holds, in phenomenological fashion, that emotions are constructed within and through a lived context wherein such distinctions cannot be made *a priori*. Emotions, she states, circulate and take shape as they move between bodies and objects, thereby forming the identities of those bodies and objects. That is, embodied subjects do not pre-exist (their) feelings of other bodies and objects, but emotions are shaped within and through their encounters with those objects and bodies—all the while forming and shaping those bodies and objects. In this sense, feelings are done *and* do something: they are produced in interaction with the world, and they produce the things and bodies that they “touch” in that world.

Ahmed explains how fear produces the boundary between the self and the object that entices fear—and, as such, what is considered an object of fear and a subject to begin with (Ahmed 2015). One example that Ahmed regularly returns to is an encounter between a bear and a child. In this encounter, the child trembles with fear and flees. Ahmed argues that in this situation, the bear *in itself* is not fearsome—but it is *to* the child. Fear and fearsome objects and subjects, in other words, are relational: “So fear is not in the child, let alone in the bear, but is a matter of how child and bear come into contact” (Ahmed 2015, 7). This encounter entails attributing the child’s (the subject’s) feeling of fear toward the bear (the object), “which moves the subject away from the object” (Ahmed 2015, 8). Fear, in this sense, is relational and performative—it is doing something, it produces bodily reactions and (increased) spaces between bodies and objects—and it is also ontological—it shapes beings as, for example, vulnerable and dangerous.

This ontological (relational) performativity of fear has an inherent embodied, cultural, and temporal dimension. After all, in the encounter between subjects and objects of fear, the latter makes an impression on the subject’s *body* and evokes a *bodily* response. The child trembles and runs, thereby increasing the space between body and object and co-constructing what is a vulnerable subject and a dangerous object. Besides this embodied dimension, there is also a complex temporal-cultural dimension at play in fear. The child’s fearful reaction is arguably not (just) instinctual or based on the child’s own experience with bears; rather, it (largely) comes from the image of the bear to be feared, created through cultural imaginaries and collective, historical memories. Before ever actually encountering a bear, the child already knows, on a bodily level, that it needs to be afraid of bears. Ahmed states that even when—or perhaps exactly because—the fearsome object is not present (yet), “we already have an impression of the risks of the encounter, as an impression that is felt on the surface of the skin” (Ahmed 2015, 7). What is more, the bear may even be more fearsome in such imaginations—when it is not actually present. Consequently, Ahmed holds that the emotion of fear does not increase with the actual presence of its object. This means that when the object of fear is not a (future) possibility anymore but an actual presence, the fear of it does not expand. Instead, it is its temporal rapprochement that causes intense fear:

Fear’s relation to the object has an important temporal dimension: we fear an object that approaches us. … Fear projects us from the present into a future. … So the object that we fear is not simply before us, or in front of us, but impresses upon us in the present, as an anticipated pain in the future. (Ahmed 2015, 65)

Hence, rather than a present object of fear (for example, an encounter with a bear but also being ill or experiencing actual pain), it is a future development, a projection, an anticipated bear, illness, or pain that causes fear. “Fear,” she summarizes, “responds to what is approaching rather than [what is] already here” (Ahmed 2015, 65).

Understanding the performative and ontological, as well as the indivisibly connected embodied, cultural, and temporal dimensions of fear, is helpful in examining the meaning of dementia self-testing apps. With this conceptualization, it is possible to reveal how taking a digital self-test for dementia leaves an impression: it may shape not only the experience of fear but also what is feared and who is fearful. Fearful responses in the context of self-testing for dementia, so we argue, should not be approached as individuated psychological reactions or as collective dispositions but rather as complex relational encounters that co-shape the experience of self-testing and fearful bodies and fearsome objects.

## Phenomenological Analysis of Fear in Dementia Self-Testing

In examining the lived meaning of self-testing for dementia and how it may entice fear in users, this section discusses experiences of fear that come forth in the analysis of the interviews with two users of the self-testing app for dementia. In describing and interpreting these dimensions, we used Ahmed’s phenomenology of fear as a guiding tool. This allowed us to identify structures in the interviewees’ experiences and disentangle and make sense of the complexities and ambiguities in their experiences. Our work resulted in identifying four dimensions of fear: performative, ontological, embodied, and temporal. In outlining how these fearing dimensions may be understood in self-testing for dementia, we illustrate our points with quotes from the two interviews.

### Performative Fear: Shaping Sense-Making and Taking Control

As mentioned in the section on previous research, many people are afraid of dementia (Kessler et al. 2012, 276). This may not be a surprise as dementia has a relatively high prevalence and because it has become a powerful term in our popular culture (Šestáková and Plichtová 2020). It is, moreover, not only a potentially lethal but also a bodily and socioculturally debilitating disorder. Yet, the fears described do not necessarily stop people from taking a dementia self-test. On the contrary, fear may instead function as a motivator *par excellence* for people to take the test in the first place.

Ahmed writes that fear is performative; it *does something.* Indeed, a fear of dementia may also press upon the user-subject with a certain effect: a felt need to take the test. Both interviewees, James and Andrew, reference explicitly and implicitly in their interviews that they fear having dementia. At the same time, they are quite resolute and do not question that they should take the test. They elaborate:

From my standpoint, I consider it just one of the things I have to do, going to my doctor. That’s my job. I have to take the test. (James)

I feel a little nervous about it. I didn’t use to but as the Parkinson’s was diagnosed, umm, then, it’s just harder to draw the clock and… anyway, I do it. (Andrew)

Despite feeling nervous about the test, James and Andrew see the test as just “a thing they have to do.” This imperative may be fueled by the priority that early detection of dementia has been made out to be in the medical context. The need to know about one’s cognitive health may also be rooted in a need to make oneself and one’s world understandable to others and oneself. In this sense, the test helps with acquiring a better sense of oneself for oneself and others. But significant for this study is that within and through those increased self-understandings, one may try to take control over and actively shape the way in which we are as embodied beings in the world. Fear of dementia and the subsequent increased self-knowledge through self-testing, then, may sustain, foster, or even enhance this need to take control through that knowledge: to—hopefully—avert the dreaded possibility of having dementia or at least to minimize its debilitating consequences. The test, in this sense, may be understood as not only increasing self-knowledge but also as a way to possibly take (more/better) control over a fearsome future possibility.

Understanding fear of dementia as performative thus allows us to explain how fear increases the need for control and self-understanding, which creates the perceived necessity of taking a test. This way, the combination of fear and technology may constitute a tool of responsibilization. Importantly, this motivational role of fear also has an economic dimension. It is, as Fox and colleagues put it, “quite possible that [a] considerable ‘market’ can be generated through capitalization of fear of dementia and cognitive decline” (Fox et al. 2013, 511). The commercial dementia self-testing app may also capitalize on the fear of dementia. In fact, the app’s web presence and introduction video help generate concerns about cognitive health and, in doing so, help introduce a problem to which the app offers a convenient solution.

#### Ontological Fear: Ab/Normal Selves and Un/Rightful Positionings

As we have already described in the previous section, the performative aspect of emotion has ontological implications as it may help fearsome subjects take control in searching for and shaping self-understandings. For Ahmed, however, the ontological performativity of emotions is (also) more fundamental and, especially in fear, more perilous. That is, as emotions reside in and move between subjects and objects, they always already form them—they shape what is considered a “me,” a “we” or what is an “inside,” and by implication, what is a not—“me,” a “them,” or an “outside.” In forming a (group) identity, emotions make use of and constitute normative notions of inclusion and exclusion. In defining what is a “we” and a “they”, emotions define what is and is not a rightful (good, normal) subject over and against illegitimate (bad, abnormal) others (Ahmed 2015, 66): fear makes the child understand itself as “me,” the inside, threatened by the “not-me,” the bad bear. Given these normative identity politics in emotions, fear is, for Ahmed, an example of how such identity politics become particularly risky. She argues that fear “works by establishing others as fearsome insofar as they threaten to take the self in”—but not just the self as an entity, rather a threat “to one’s very life, to one’s very existence as a separate being with a life of its own. … Fear might be concerned with the preservation not simply of ‘me,’ but also ‘us,’ or ‘what is,’ or ‘life as we know it,’ or even ‘life itself’ ” (Ahmed 2015, 64).

This fundamental ontological dimension of fear may also be at work, in various ways, in the case of self-testing for dementia. For James, for instance, the test was crucial in determining whether he could continue his work as a medical doctor—a role he profoundly identifies with. The test ties together his fears of losing his identity as a doctor, his social relationships, and a culture wherein he, as a doctor, may have a (rightful) place.

In both Andrew’s and James’s narration, we see other hints of how their fear of dementia self-testing may amount to a fear of not being normal and not being a rightful self in the world. They both fear “failing the test”: to either not get the result they deserve or not have the perfect score they desire. They are also very relieved when it turns out that they filled out (parts of) the test correctly. Such a result, for Andrew, did not only feel good, but he even felt “accomplished.” The anxiety over losing points, the desire to score correctly, and the feeling of relief or even accomplishment point to the significance of this test. Fear, in itself ontologically performative and concerned with a loss of self, is here directed at a disorder that also entails a strong culturally formed negative image connected to the previously addressed loss of selfhood and identity. Indeed, in shared, everyday beliefs as well as in various media outlets such as films and novels, people with dementia (and MCD) are not only regarded as abnormal selves—as (becoming) “an idiot” (Husband 2000), even as non-people. No, these normative social imaginaries of dementia show that much of what we regard as “normal” people or people to begin with is dependent on our cognitive abilities. More than that, such imaginaries help to designate people with dementia as non-normal; they often also portray them as a threat to society. Dementia has been represented as “a plague” and a “silent tsunami” (Zeilig 2014), suggesting that it infects society as a whole and washes away life as we know it. Considering these imaginaries, it is not surprising that Andrew and James seem to attach so much value to the result of the test and feel accomplished when they get a satisfactory test. The result, for them, averts the threat that they are or become abnormal and that they lose their rightful place in the world.

#### Embodied Fear: Intersectional Bodies

While social imaginaries of dementia may be largely shared in our culture, Ahmed’s theory does not entail that every body experiences fear of dementia the same way. She stresses that fear is an inherently embodied and personal experience, wherein one’s socio-cultural positioning may be at play but is not deterministic for how one experiences fear. That is, social imaginaries like those of people with dementia as “idiots” may inform seeking control to avert a dreadful lived future with dementia and go to a doctor’s office to take a self-test. But while these shared social imaginaries of dementia help to shape whether and how this disease and a possible future diseased self is identified as fearsome, it does not mean that everybody experiences fear of dementia in the same way—even that dementia is identified as an object of fear to begin with. A person’s particular embodiment (their gender, age, dis/abilities, etc.), which is imbued by meaning within and through the particular socio-cultural context that person lives in, has an effect on how dementia and a future demented self are experienced as fearsome (or not) (Laforce and McLean 2005, 205; Suhr and Kinkela 2007, 225).^5^ In understanding what fear means, then, an intersectional perspective is pivotal—or as Ahmed would formulate it: it is pivotal to understand how “fear is felt differently by different bodies” (Ahmed 2015, 68).

Fear is a thoroughly embodied experience. Just as the child trembles with fear when encountering the bear, James stutters when talking about his fear of dementia; both James’s and Andrew’s fears do not seem to make them flee from a self-test but rather motivate them to take it. Ahmed elaborates on this embodied fear as follows:

[T]he feeling of fear presses us into that future as an intense bodily experience in the present. One sweats, one’s heart races, one’s whole body becomes a space of unpleasant intensity, an impression that overwhelms us and pushes us back with the force of its negation, which may sometimes involve taking flight, and other times may involve paralysis. (Ahmed 2015, 65)

When we turn to the narratives of James and Andrew, we may begin to see how fear may be experienced differently by different bodies in different lived contexts—even though neither their bodies nor their lived contexts are arguably that (categorically) different from each other. James and Andrew are both white, middle-aged, high middle-class, heterosexual men living in the United States with good access to healthcare.^6^ Andrew, however, reflects on how his experienced ability to successfully finish the tasks of the dementia self-testing intersects with the physical inabilities he has as a result of his Parkinson’s disease. For Andrew, the tremor in his fingers made it harder to draw the clock on the tablet. He elaborates:

I remember going at the clock and how, well, of course, the thing is, I have Parkinson’s and depends on which time of the day and medication, the score will vary. Sometimes my writing looks really good and actually, thank God, most of the time, it looks readable and you know, not wiggly. So I get, you know, but I mean if you catch me towards the end of the… when the medication is starting to wear off, that doesn’t happen all the time, but sometimes, then the fingers get shaky. (Andrew)

The material actuality of the technology and Andrew’s Parkinson’s diagnosis contribute to his fear of “failing” the test and, as a result, being diagnosed with cognitive impairments. Andrew’s Parkinson’s, in other words, may have increased his feeling of vulnerability, which made him perceive the test more fearfully.

James, in his interview, also reflects on his particular embodied state and diagnosis with another illness and how this influences his fear of taking a dementia self-test. Rather than a catalyzer of his dementia fears, however, his diagnosis with ADHD seems to serve as a protection against someone claiming he has cognitive impairments because of MCD or dementia. He argues that what others might interpret as a sign of MCD or dementia—being forgetful or having difficulties following conversations—he can explain as the symptomatic appearance of ADHD. His ADHD diagnosis, then, seems to make him anticipate the dementia self-test with a more relaxed attitude.

These examples show not only that fear in self-testing for dementia is itself an extended embodied experience but also that it incorporates other bodily aspects (such as physical consequences of other diseases). These sedimented, past experiences shape whether and to what extent a user experiences the test as fearsome. Attending to a user’s situatedness is, therefore, crucial to understanding what the self-testing app can mean for them.

### Temporal Fear: Tangible Future Selves

Besides the impact self-testing may have on the various kinds of bodies, the anxious anticipation of Andrew and James to get their test result also reveals that fear in dementia self-testing has a significant temporal dimension: it can be understood as a response to an *approaching* fearful object. Testing for dementia, in its very idea, is a way to foreground and explicate the possibility of a fearsome future—which, in the case of dementia, may include (mental and physical) pain and becoming (more) forgetful and less capable. This foregrounding happens explicitly with the case app when the results are explained to the user by a doctor in a personalized video. In James’s narration of using the dementia self-testing app, the future possibility of developing and having cognitive problems seems to play a pivotal role:

James: I don’t think I got MCI [Mild Cognitive Impairment] but I may be wrong! So ev—every time I see my doctor [to take the test], have I slipped? [laughs] ok?

Alexandra: So there is a little bit of…

…

James: Anxiety. There is anxiet— A little bit of anxiety, a little bit of concern.

James explicitly states that every time he takes the test, he is “anxious” about the possibility that he “slipped” (i.e., lost [part of his] cognitive abilities). The use of the metaphor “slipping” already reveals how he positions himself over and against this possible and unwanted future: he seems to regard himself as possibly already on the “slope” (down) towards a lived future reality of having dementia.^7^ In this sense, the dementia self-test seems to make the future frightening possibility of having dementia more tangible and real through the test and the numerical score the users receive. When taking the test, users may imagine and position themselves explicitly on the way toward having dementia. Fear, then, of the encounter between user-subject and test-object is not just performative in the sense that it creates a fearful subject and a fearsome object, but it also is performative in that it aligns the user with people at risk for dementia.

It is important to address here that in dementia self-testing, the fearsome object is neither simply the condition of dementia itself nor the materiality of the self-testing app. Taking the insights in this section and the section on ontology together, we can show that what is interesting about the case of dementia self-testing is that the fearsome object is also *my own embodied self in the future*. The fearful object in dementia self-testing, in other words, is a future self that threatens not only oneself in its present form but also its current relationships and its rightful place in the world. Fear of dementia, in this sense, creates a “future me,” a self with dementia that has lost its identity, relationships, and positionings, which the “I,” the present self, is positioning itself over and against. This future fearsome self, then, is arguably made more tangible and real as a dreaded possibility in dementia self-testing. Within and through taking this test, after all, a concrete encounter with this future self is created, especially in the case of a positive test result that is explained in the personalized test result video.

## Conclusion

In this article, we developed four different dimensions of fear in the context of dementia self-testing based on Ahmed’s conceptualization of fear as a complex, performative encounter between embodied selves and approaching objects of fear (Ahmed 2003, 2015). First, fear does things, namely motivating us to take a self-test and to try to take control over our health. Second, fear in self-testing for dementia shapes (and is shaped by) the ways in which we make sense of ourselves and others as, for example, cognitively deficient. Third, fear in dementia self-testing constructs (and is constructed by) our differently embodied presence in the world: fear influences the ways in which we experience and deal with that tremor caused by Parkinson’s or that forgetfulness because of ADHD.

Finally, what we sketched out as perhaps the most far-reaching implication of fear in the context of dementia self-testing is that fear may explicitly turn inward, toward ourselves. That is, in fearing dementia, we may fear a future (possible) self—a cognitively deficient one—that replaces our current self. In dementia, we may even say we fear a loss of the self in the future. This turn to a future self may be heavily mediated by self-testing technology and practice. The practice of self-testing, we argue, may make this fear of a future self or this fear of self-annihilating more tangible in foregrounding and explicating the possibility of becoming (more) cognitively deficient. Being afraid of “slipping” toward having cognitive impairments is co-constituted through the practice and outcome of self-testing: the test results may explicate that having (to deal with) a certain new, unwanted future of cognitive impairment is likely (or not). This fearful test result, in turn, is made particularly tangible not only in an overview of the false and correct answers but also in a personalized video wherein these results and the accompanying (imagined) futures are explained.

In exploring these temporal and other dimensions of fear (performative, ontological, embodied), this article shows that fear in the context of self-testing for dementia should not be approached as merely an individual psychological reaction or as only a collective, sociological disposition (Ahmed 2015). It also shows that there is more to fear in self-testing than being afraid of getting the wrong results (Sarkar et al. 2016) or of not being taken seriously by doctors (den Oudendammer and Broerse 2019). We demonstrated the relevance of fear by conceptualizing it as an intersubjective, culturally situated, and embodied encounter between selves, others, and objects that shape and construct dementia self-testing experiences, as well as the involved fearful bodies and fearsome objects.

With the ever-more increased implementation of self-testing apps in professional health institutions and the expanded accessibility of such apps by individual (potential) users, the findings of this paper allude to the importance of discussing the ways in which we should (or should not) deal with such apps in the context of not only dementia care but also other illness and health contexts. While there are many—and perhaps rightly so—celebratory voices surrounding the popularization of self-testing apps, this article also shows that these tests are not just quick and easy tools (Ruggeri et al. 2016) and that caution should be warranted when recommending and using self-testing apps for dementia. After all, self-tests may capitalize on someone’s fear of a certain illness, even for a future self, which may lead to considerable additional (mental) distress and decreased well-being. Especially for the group of the so-called “worried well” (i.e., people who are cognitively healthy and look for reassurance) (Royal College of Surgeons of England 2018), waiting for the test result or even a positive test result could entail a significant confrontation with such an unwanted future, as they might not have entertained the possibility of having cognitive problems. Taking into account such “side effects,” it seems defensible for policymakers and health care professionals to have a certain hesitancy when recommending using self-testing apps. Moreover, when actually using self-testing apps, users may benefit from being extensively guided in the process. That is, listening to users’ possible fears and providing them with tailored advice and knowledge (about the testing process, the results, the disease, and possible treatments) may help them ease and deal with their fears. Systematic qualitative interview studies that take into account the complex performativity of fear in dementia self-testing apps are needed to further discern what fear means in this self-testing context and how the experience of dementia self-testing could be improved.

## Data Availability

In accordance with research ethics regulations, the audio files and transcripts of the interviews are not available to the public.
